# Distribution of Neuropeptide F-Like Immunoreactivity in the Eastern Subterranean Termite, *Reticulitermes flavipes*

**DOI:** 10.1673/031.008.6801

**Published:** 2008-11-03

**Authors:** Andrew B. Nuss, Brian T. Forschler, Joe W. Crim, Mark R. Brown

**Affiliations:** Department of Entomology, University of Georgia, Athens GA

**Keywords:** immunocytochemistry, nervous system, midgut

## Abstract

The nervous system and gut of worker, soldier and alate castes of the eastern subterranean termite, *Reticulitermes flavipes* Kollar (Isoptera: Rhinotermitidae) were examined for immunoreactivity to an antiserum to *Helicoverpa zea* (Boddie) (Leipidoptera: Noctuidae) MP-I (QAARPRF-NH_2_), a truncated form of neuropeptide F. More than 145 immunostained axons and cell bodies were seen in the brain and all ganglia of the ventral nerve cord. Immunoreactive axons exiting the brain projected anteriorly to the frontal ganglion and posteriorly to the corpora cardiaca and corpora allata. In the stomatogastric nervous system, immunoreactive axons were observed over the surface of the foregut, salivary glands, midgut and rectum. These axons originated in the brain and from 15–25 neurosecretory cells on the foregut. Staining patterns were consistent between castes, with the exception of immunostaining observed in the optic lobes of alates. At least 600 immunoreactive endocrine cells were evenly distributed in the midguts of all castes with higher numbers present in the worker caste. Immunostaining of cells in the nervous system and midgut was blocked by preabsorption of the antiserum with *Hez* MP-I but not by a peptide having only the RF-NH_2_ in common. This distribution suggests NPF-like peptides coordinate feeding and digestion in all castes of this termite species.

## Introduction

The invertebrate neuropeptide Fs (NPFs) are members of a neuropeptide family that includes three related vertebrate peptides, neuropeptide Y (NPY), peptide YY (PYY), and pancreatic polypeptide (PP) (Brown et al. 1999; Berglund et al. 2003; Pedrazzini et al. 2003; Conlon and Larhammar 2005). Both NPFs and the NPY-related peptides are encoded by homologous genes and processed from propeptides into a bioactive peptide with 36 to 40 amino acids and a Phe or Tyr-NH2 carboxy (C-) terminus. The first insect NPF was isolated from *Drosophila melanogaster* (Brown et al. 1999) and later, a related one was isolated from the mosquito, *Aedes aegypti* (Stanek et al. 2002). Since then, other insect NPFs have been identified by bioinformatics in the genome databases of *Anopheles gambiae* (Garczynski et al. 2005), and *Apis mellifera* (Hummon et al. 2006), all of which are holometabolous species. To date, NPF has been identified by bioinformatics from only a single hemimetabolous species, *Locusta migratoria* (Clynen et al. 2006). Truncated forms (8 to 10 amino acids) of apparent NPFs have been isolated from the corn earworm, *Helicoverpa zea* (Huang et al. 1998) and the desert locust, *Schistocerca gregaria* (Schoofs et al. 2001).

Immunocytochemical studies of insects provided the first evidence for vertebrate-like peptide hormones in insects. In particular, studies using PP or NPY antisera showed that immunoreactive peptides were localized in specific cells in the nervous system and midgut of cockroaches, crickets, locusts, flies and moths (El-Salhy et al. 1980; Iwanaga et al. 1981; Duve and Thorpe 1982; Endo and Nishiitsutsuji-Uwo 1982b; El-Salhy et al. 1983; Myers and Evans 1985; Brown et al. 1986; Iwanaga et al. 1986; Schoofs et al. 1988). In hindsight, the objective of identifying a gut-specific NPF from an insect was attained with the isolation of the two midgut peptides, *Hez* MP-I (Table 1) and -II, from corn earworm larvae, *Helicoverpa zea* (Huang et al., 1998). Their chromatographic purification was driven with a radioimmunoassay (RIA) using an Arg-Phe-NH2 antiserum. Subsequently, an antiserum specific to *Hez* MP-I was produced and used in RIAs to monitor isolation of the *D. melanogaster* NPF (Brown et al. 1999). Using immunocytochemistry, the *Hez* MP-I antiserum showed that the NPF was present in relatively few brain neurons and neurosecretory cells and in many midgut endocrine cells in *D. melanogaster* larvae and adults. In vertebrates, the NPY-related peptides also display expression as a brain-gut axis that regulates feeding behavior and digestion (Neary et al. 2005; Cox 2007). Recent studies show that NPFs also affect feeding and digestion in insects. For mosquito larvae, NPF inhibits peristalsis and ion transport of the midgut in vitro (Onken et al. 2004). In *D*. *melanogaster,* alterations in the gene for NPF and its receptor are associated with specific feeding and food-searching behaviors of larvae particularly under food-deprived conditions (Shen and Cai 2001; Wu et al. 2003; Wu et al. 2005; Lingo et al. 2007).

**Table 1.**
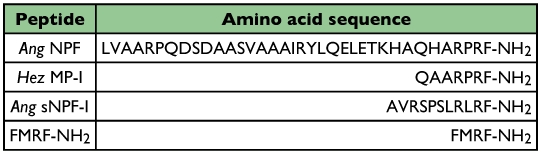
Amino acid sequence of peptides used to preabsorb Hez MP-I antiserum.

As a first step to determine whether an NPF exists in termites and if it affects feeding, *Hez* MP-I antibody was used to map and compare the distribution of NPF-like immunoreactivity in the brain, ventral nerve cord and alimentary tract of alate, worker, and soldier castes of the eastern subterranean termite, *Reticulitermes flavipes* (Kollar) (Isoptera: Rhinotermitidae). Different feeding behaviors have been observed among castes of this species. The worker caste feeds directly on wood or other cellulose-rich substrates, whereas the soldier and alate castes rely on food shared by the workers (Laine and Wright 2003). To date, only three peptide hormones have been isolated and structurally characterized from termites: *Zootermopsis nevadensis* diuretic hormone (*Zoone* DH) (Baldwin et al. 2001), *Microhodotermes viator* corpus cardiacum peptide (*Miv*-CC) and *Periplaneta americana* cardioaccelerating hormone (*Pea*-CAH-I) from *Mastotermtes darwiniensis* and *Trivervitermes trinervoides* (Liebrich et al. 1995). The immunocytochemical distribution of pigment-dispersing hormone was reported in the central nervous system of *Neotermes castaneus* (Sehadova et al. 2003) and *Dippu-*allatostatin-like immunoreactivity in the brain of *R. flavipes* (Yagi et al. 2005). Termites are economically important structural pests worldwide (Su and Scheffrahn 2000), and antagonists of peptide hormones could be used to disrupt the internal signaling systems of pest insects as a potential management tool (Orchard et al. 2001; Gade and Goldsworthy 2003) providing an alternative to the chemistries currently used for termite management.

## Materials and Methods

### Animals

*R. flavipes* were collected from 12 different termite-infested logs in Whitehall Forest south of the University of Georgia campus. Workers and soldiers were attracted from logs into PVC tubing containing moistened, rolled cardboard. Termites were transferred to plastic boxes containing moistened slats of pine wood and filter paper and stored at 24°C in total darkness. Workers and soldiers used for immunocytochemistry were taken between 7–66 days after collection. Alates were captured as they emerged from logs in the lab during this species' swarming season (January through April). Alates were either used within one to two days after emergence or male and female pairs were placed together in nesting material for 6 days before use.

## Morphological Measurements

The brain and ventral ganglia of alates (n ≥ 2), soldiers (n ≥ 6) and 4th instar or larger workers (n ≥ 5) were dissected in phosphate buffered saline (PBS), and mounted on microscope slides. Images were taken with a digital camera (JVC America Corp., model KY-F70BU, www.jvc.com) mounted on an Olympus BX60 microscope (www.olympus.com) using Auto-Montage Pro software (Synoptics Ltd., version 5.01.0005, 2004, www.synoptics.co.uk). Auto-Montage Pro was used to take measurements of length and width for each ganglion and length of ganglial connectives. Diagrams of the caste nervous systems were created from digital images of representative ganglia that were traced in Adobe Photoshop CS (Adobe Systems Inc., version 8.0, 2004, www.adobe.com) for each caste examined.

Alimentary tracts of workers (n ≥ 6) and soldiers (n ≥ 4) were dissected, mounted and photographed as above. Tissues were partially unfolded so that the entire alimentary tract could be observed. Length and width of each gut region were measured with Auto-Montage Pro. A generalized diagram of the *R. flavipes* alimentary tract was created from digital images for a description of immunocytochemical data.

### Whole Tissue Immunochemistry

Brain, ventral nerve cord and alimentary tract were dissected from alates, soldiers and 4th instar or larger workers in 4% paraformaldehyde/PBS solution (pH 7.4). Tissues were fixed for 1 h in the paraformaldehyde solution at 4 °C and then dehydrated in 15 min washes of ethanol/PBS solution in the following series: 30, 50, 70, 100%, and rehydrated in PBS/70% ethanol for 15 min, and PBS for 15 min. Goat serum (5%) with 0.1% Tween 20 (PBS-GS-T) was used to block tissues for 1 hour. *Hez* MP-I antibody (35B, Huang et al. 1998) was diluted 1/800 in PBS-GS-T and incubated with tissues overnight at 4 °C. Tissues were washed with PBS-GS-T (3 × 30 min) and then incubated with goat-anti rabbit Alexa Fluor 488F® (Molecular Probes, Eugene, OR, USA, 1:2000 in PBS-GS-T, www.probes.com) overnight. Finally, tissues were washed with PBS-T (3 × 30 min), and then mounted on glass slides in a 1:1 PBS/glycerol solution. Controls were performed by preabsorbing primary antibody with FMRF-NH2 (3.3 × 10^-5^ M), *Hez* MP-I (7.3 × 10^-5^ M), *Ang* sNPF I (6.3 × 10^-5^ M), or *Ang* NPF (1.2 × 10^-4^ M) (Table 1) for 24 h prior to tissue incubation or by omitting the primary antibody. Specimens were viewed and photographed using the same microscope equipped with epifluorescent optics mentioned above, and at least three of the antiserum and control-treated tissues from individuals of the different castes were observed for the results reported herein.

### Transmission Electron Microscopy

Midguts from worker *R. flavipes* were dissected and fixed according to the procedure of Grube et al. (1997). Briefly, worker midguts were fixed in 2% glutaraldehyde / 0.1 M cacodylate buffer solution (pH 7.0) for 20 h at 4 °C. A cacodylate buffer rinse was followed by a secondary fix in 1% OsO_4_ (1 h at 4 °C). Tissues were washed in 0.1 M cacodylate buffer (15 min at 4 °C), H_2_O (2×, 15 min at 4 °C), ethanol (30, 50, 70, 95, 100, 100, 100%, 15 min each, 4 °C), and propylene oxide (15 min at 4 °C). Midguts were embedded in Epon 812 resin and polymerized overnight at 65 °C. Sections (∼40 nm) were stained with uranyl acetate and lead citrate and photographed with a JEOL 100CX II transmission electron microscope.

## Results

### Nervous system - morphology

**Figure 1.**
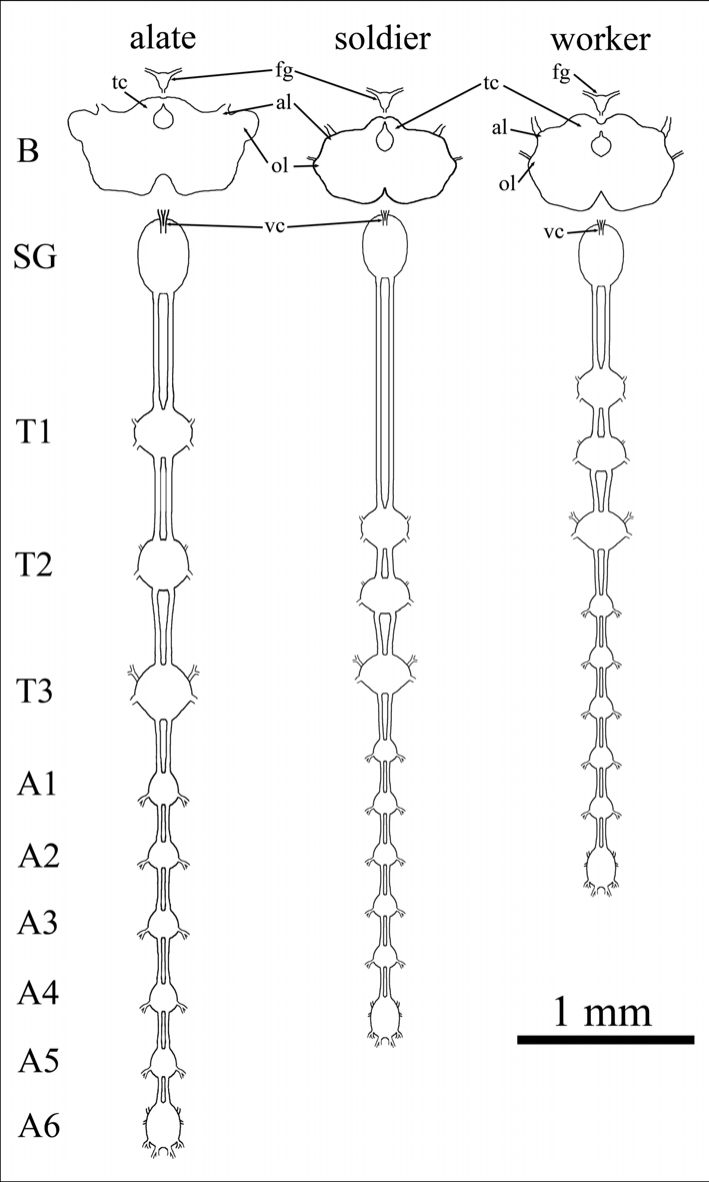
Dorsal view of the brain and ganglia of *Reticulitermes* *flavipes* alates, soldiers and workers. In this figure brains are displaced to show the subesophageal ganglion located ventrally. Both the ventral connective of the subesophageal ganglion and the frontal ganglion connect to the tritocerebrum of the brain. Abbreviations: al, antennal lobe; fg, frontal ganglion; ol, optic lobe; tc, tritocerebrum; vc, ventral connective; B, brain; SG, subesophageal ganglion; T 1–3, thoracic ganglia; A 1–6, abdominal ganglia.

The central nervous system in the three castes of *R. flavipes* is comprised of a brain, frontal ganglion, subesophageal ganglion (Thompson 1916), three thoracic ganglia and six abdominal ganglia joined by parallel axon tracts (Figure 1). The first five abdominal ganglia are nearly identical in size, but the sixth, a fusion of five terminal ganglia (Richard, 1969), is larger (Table 2).

The brain and ventral ganglia of the alate caste were larger than those of workers and soldiers (Table 2). Alate brains had well developed optic lobes compared to the reduced optic lobes of soldiers and workers. The subesophageal ganglion was similar in size between soldiers and alates but smaller in workers. Brain and ventral ganglia proportions were similar in soldiers and workers, but ganglial connectives between the subesophageal ganglion and the first thoracic ganglion were approximately twice as long in the soldiers than those of workers or alates to accommodate the longer head length.

**Table 2.**
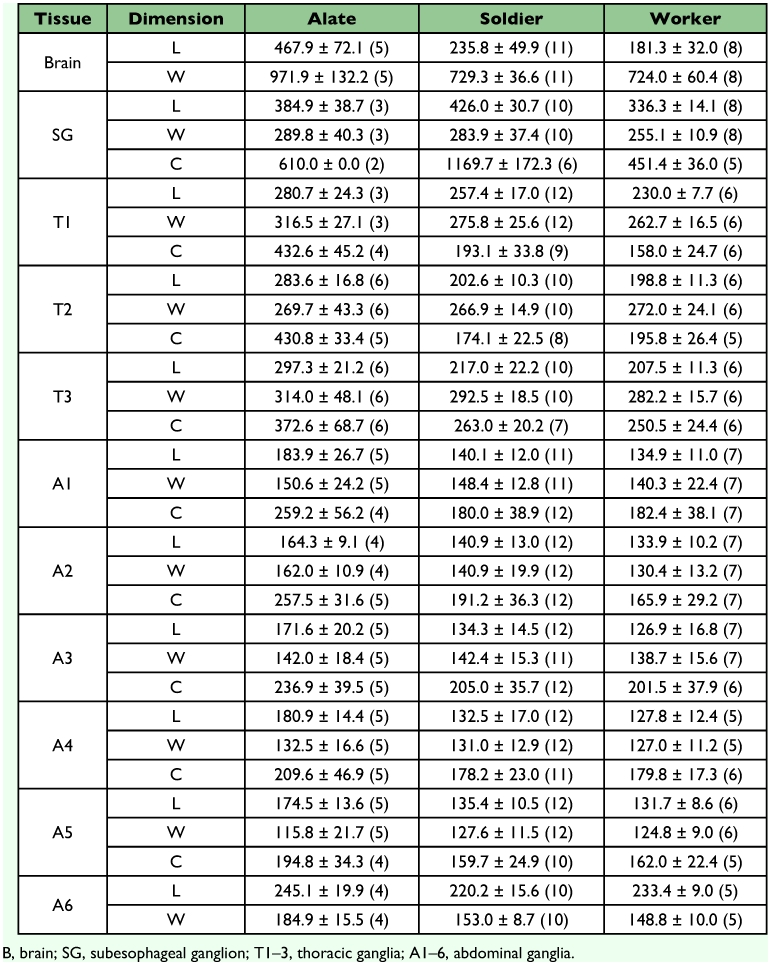
Average length (L), width (W) and length of ganglial connectives (C) of dissected brains and ventral nerve cords of the alate, worker and soldier castes of R. flavipes (±SD *µ*m). Number of specimens measured is included in parenthesis.

### Brain and frontal ganglion

**Figure 2.**
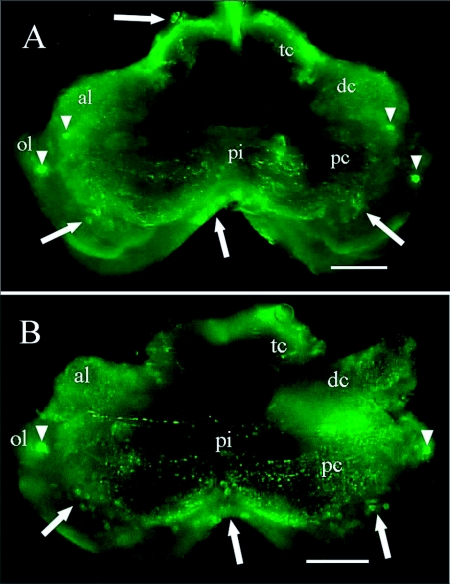
NPF-like immunoreactivity of the brain of a soldier (A) and worker (B) of *Reticulitermes* *flavipes.* Clusters of immunoreactive cells (arrows) and brightly staining cells (arrowheads) are indicated. Abbreviations: al, antennal lobe; dc, deutocerebrum; op, optic lobe; pc, protocerebrum; pi, pars intercerebralis; tc, tritocerebrum. Bars = 100 ***µ*m.**

**Figure 3.**
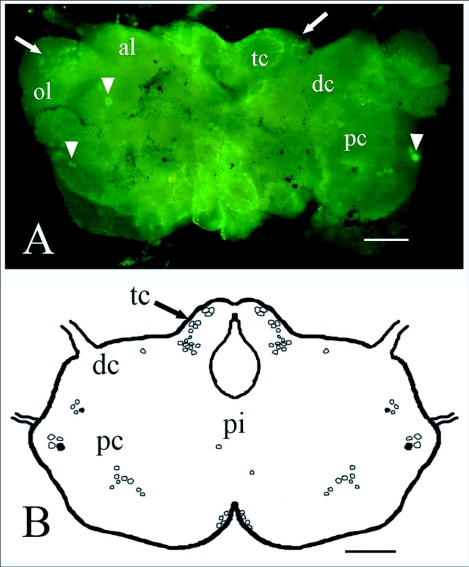
(A) NPF-like mmunoreactivity of the female alate brain of *Reticulitermes* *flavipes.* Clusters of immunoreactive cells (arrows) and brightly staining cells (arrowheads) are indicated. (B) Diagram of immunoreactive cells observed in the brain of a soldier. Clusters of immunoreactive cell bodies occurred in the same regions of alates, workers and soldiers. Brightly immunostaining cell bodies are indicated in black. Abbreviations: al, antennal lobe; dc, deutocerebrum; ol, optic lobe; pi, pars intercerebralis; pc, protocerebrum; tc, tritocerebrum. Bars = 100 *µ*m.

Immunoreactive cells were observed in all regions of the central nervous system of the three castes examined, except for the frontal ganglion. In all castes, strong immunostaining was observed in a pair of neurosecretory cells in the protocerebrum and another pair on the anterior distal margin of the protocerebrum (Figures 2, 3).

Immunoreactive cell bodies in the brain were counted for each caste (Table 3). On average, approximately 70 immunoreactive cells were counted per brain and no significant differences in number of cells were found between castes (*F* = 0.03; df = 2; *P* = 0.967). The number of immunoreactive brain cells varied widely between individuals of a caste. For soldiers the range was 32 to 117 immunoreactive cells and for workers 31 to 94 immunoreactive cells. The proportion of immunoreactive cells in various brain regions was similar within a caste (Table 4). Many immunoreactive cells were observed in the tritocerebrum (43% of the immunoreactive brain cells in soldiers and 25% in workers) and in the protocerebrum (30% in soldiers and 51% in workers) (Table 4, Figures 2, 3). In alate brains, immunoreactive axons were observed within the optic lobes (Figure 3A) but not in those of workers or soldiers.

**Table 3.**
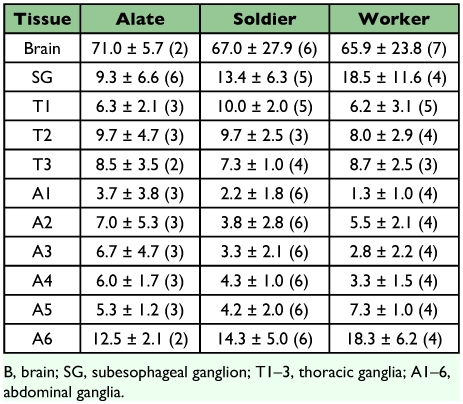
Average number of immunoreactive NPF-like cells counted in brain and ventral nerve cord of alates, soldiers, and workers (±SD). Number of specimens counted is included in parenthesis.

**Table 4.**
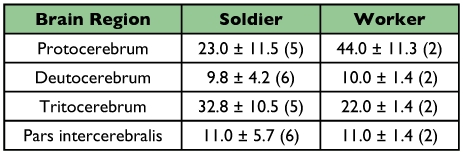
Average number of NPF-like immunoreactive cell bodies counted by brain region in soldiers, and workers (±SD). Number of specimens counted is included in parenthesis.

**Figure 4.**
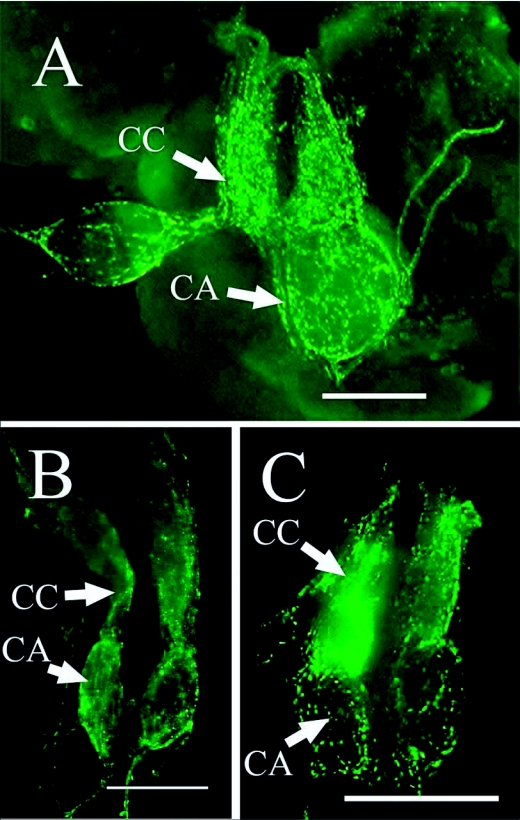
NPF-like immunoreactive axons associated with the corpora cardiaca (CC) and corpora allata (CA) of an alate (A), soldier (B) and worker (C) *Reticulitermes* *flavipes.* Bars = 100 *µ*m.

Immunoreactive axons were observed on the surface of the corpora cardiaca and the corpora allata of all castes (Figure 4), but no immunoreactive cell bodies were observed. The corpora allata of alates were larger and more rounded than those of workers or soldiers.

### Ventral nerve cord

**Figure 5.**
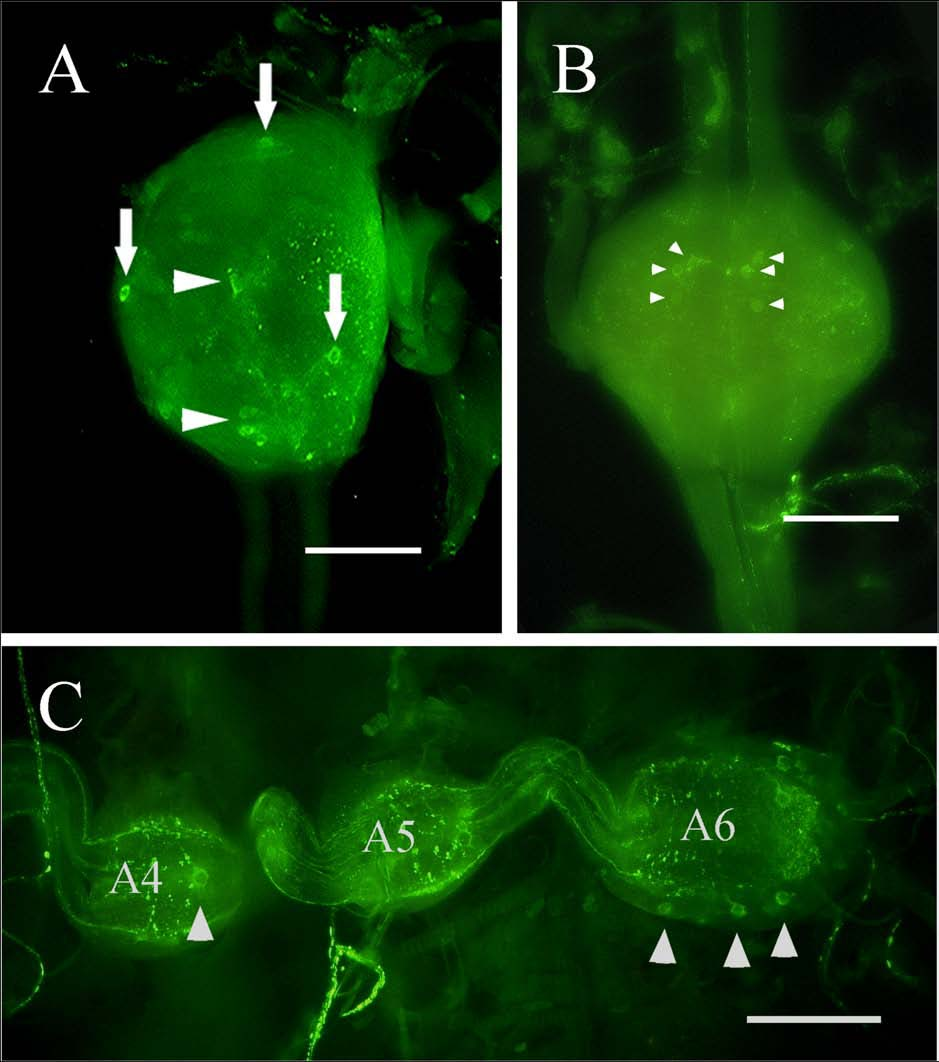
NPF-like immunoreactivity of ventral nerve cord ganglia of *Reticulitermes* *flavipes.* (A) Ventral view of subesophageal ganglion. Bright staining cells (arrows) and additional immunoreactive cells (arrowheads) were observed. (B) Thoracic ganglia. 3 pairs of brightly stained cells were observed consistently (arrowheads). (C) Posterior abdominal ganglia (A4 – A6) with scattered immunoreactive cells (arrowheads). Bars = 100 *µ*m.

In all castes, three brightly stained cells were consistently seen on the ventral surface of the subesophageal ganglion: a pair of cells on the posterior and a single cell on the anterior surface (Figure 5A). Although variable, the total number of immunoreactive cells in this ganglion was highest in workers followed by the soldiers and alates (Table 3) but differences were not significant (*F* = 1.55; df = 2; *P* = 0.251).

The thoracic ganglia in all castes contained eight brightly stained cells: two cells on the dorsal anterior of the ganglia, and six cells, in groups of three, on the ventral center of the ganglia (Figure 5B). The total number of immunoreactive cells counted in thoracic ganglia was similar in all castes (Table 3).

Abdominal ganglia 1–5 had from one to eight immunoreactive cells (Table 3, Figure 5C), but most lacked strong immunoreactivity. Each abdominal ganglion had a single pair of immunoreactive axons that extended along the abdominal body wall, although the exact tissue innervated was not determined. The terminal abdominal ganglion contained an average of 7 brightly stained cells for all castes. No clear distribution pattern for these cells was evident. Workers had more total immunoreactive cell bodies in the terminal abdominal ganglion than soldiers, and soldiers had more than the alates, although these differences were not significant (*F* = 1.04; df = 2; *P* = 0.392) (Table 3).

### Stomatogastric nervous system

**Figure 6.**
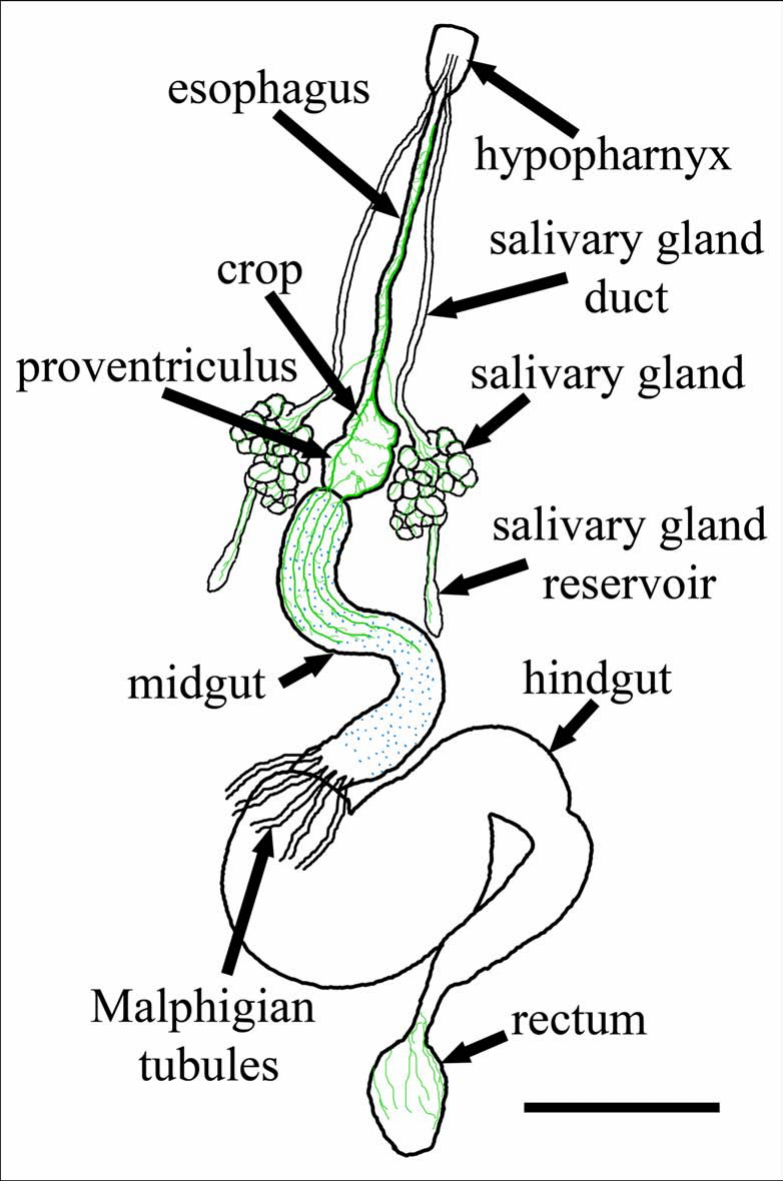
Diagram of expanded *Reticulitermes* *flavipes* alimentary tract with immunoreactive axon tracts in green and immunoreactive midgut endocrine cells in blue. Bar = 500 *µ*m.

**Figure 7.**
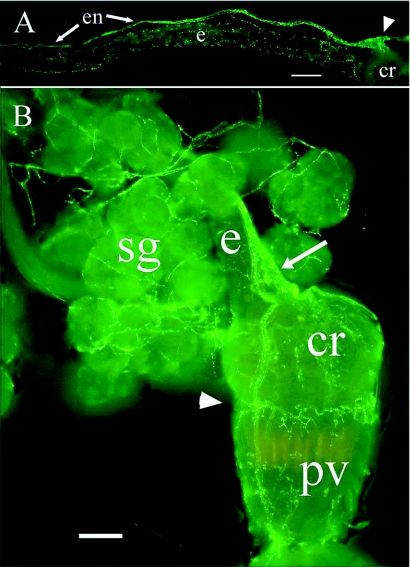
NPF-like immunostaining of the foregut of *Reticulitermes* *flavipes.* (A) Immunoreactive axons on the esophagus and immunoreactivity in the esophageal nerve. This nerve divides after the ingluvial ganglion (arrowhead) (B) Immunoreactive axons on the crop, proventriculus and salivary glands. The immunoreactive esophageal nerve enters the ingluvial ganglion (arrow) and divides over the crop. Note immunoreactive cells in the ingluvial ganglion. Branches of the nerve ring the junction of crop and proventriculus (arrowhead). Abbreviations: cr, crop; e, esophagus; en, esophageal nerve; sg, salivary glands; pv, proventriculus. Bars = 100 *µ*m.

**Figure 8.**
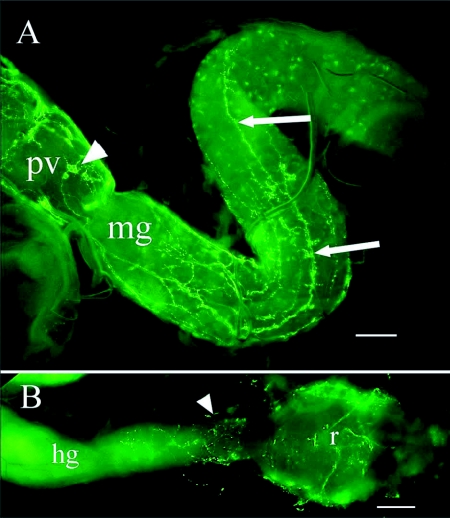
(A) NPF-like immunoreactivity of the foregut and midgut of *Reticulitermes* *flavipes.* Immunoreactive enteric plexus cell bodies on the surface of the proventriculus (arrowhead). Immunoreactive axons on the midgut surface (arrows). (B) Immunoreactive axons on the rectum and posterior hindgut/rectal junction (arrowhead). Abbreviations: hg, hindgut; mg, midgut; pv, proventriculus; r, rectum. Bars = 100 *µ*m.

Immunoreactive axons were associated with the alimentary tract (Figure 6) in all three castes. Immunoreactive axons projected to the frontal ganglion from the tritocerebrum. A single, large, immunoreactive axon tract originating in the frontal ganglion was present on the esophagus (Figure 7A). It branched over the surface of the esophagus and salivary (labial) glands and gland reservoirs (Figure 7B) and extended along the esophagus to divide over the crop after the ingluvial ganglion (Figure 7B). Five to eight immunoreactive cell bodies were in this ganglion. The axons branched over the crop and ringed the junction of the crop and proventriculus. The proventriculus was covered with immunoreactive axons, and 10 to 20 immunoreactive enteric plexus cell bodies were present on the surface (Figure 8A). The two main branches of the esophageal nerve continued down the proventriculus and branched again at the junction of the foregut and midgut. Immunoreactive axons were less numerous from the anterior to the posterior region of the midgut and ended before the hindgut (Figure 8A). Immunoreactive axons originating from the 6th abdominal ganglion were observed only on the rectum and junction of the hindgut and rectum (Figure 8B).

### Midgut endocrine system

**Figure 9.**
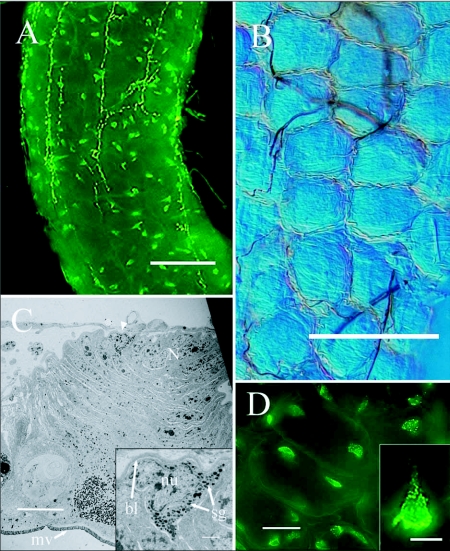
(A) Midgut endocrine cells of *Reticulitermes flavipes.* Midgut is oriented anterior (top) to posterior (bottom) Bar = 100 *µ*m. (B) Light microscopy of midgut surface showing the circular nodes. Bar = 100 *µ*m. (C) TEM of midgut cross-section showing a nidus. Midgut is oriented hemolymph side (top) to lumen (bottom). Arrowhead indicates a small portion of an endocrine cell. Bar = 10 *µ*m. Inset: Closeup of endocrine cell. Bar = 1 *µ*m. (D) Immunoreactive endocrine cells. Typically two cells occur per nidal cluster. Bar = 25 *µ* m. Inset: Closeup of pyramidal immunoreactive endocrine cell. Bar = 10 *µ*m. Abbreviations: bl, basal lamina; mv, microvilli; N, nidus; nu, nucleus; sg, secretory granules.

The midgut of *R. flavipes* is uniformly tube-like with no gastric cecae (Noirot 1995) (Figure 6, Table 5). The midgut surface is composed of circular nodes (52.2 ± 11.3 ***µ***m in diameter [n = 21]) (Figure 9B) that contain regenerative nidi and the surrounding mature columnar cells. Regenerative cell nidi were observed at the center of these nodes as revealed by transmission electron microscopy (TEM) (Figure 9C). Endocrine cells were identified by the presence of secretory granules near the basal lamina (Figure 9C inset). NPF-like endocrine cells were visualized with fluorescence microscopy using the *Hez* MP-I antibody (Figure 9A, D). These cells were clearly differentiated from axons by their characteristic bottle shape with a wide base at the basal lamina and an apical extension to the lumen of the gut (Figure 9D inset) (Nishiitsutsuji-Uwo and Endo 1981). Two immunoreactive endocrine cells were observed on opposite sides of each node (Figure 9D), but the number was not always consistent and as many as five were observed in one node.

**Table 5.**
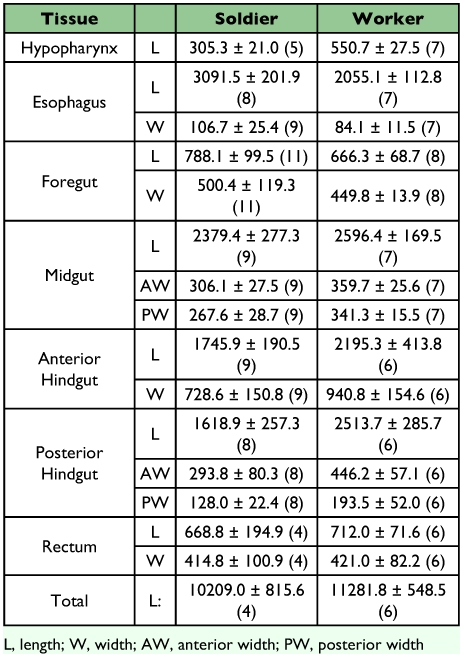
Average length (L), and width (W) of dissected gut tissues of workers and soldiers (±SD *µ*m). Widths were measured on the anterior and posterior portions of the midgut and posterior hindgut (AW and PW, respectively). Number of specimens measured is included in parenthesis.

Immunostained midguts from caste members were cut longitudinally and placed flat on microscope slides for cell counting. Immunoreactive endocrine cells were evenly distributed throughout the midgut in all castes examined. Workers had more midgut endocrine cells than soldiers or alates, soldiers had more than alates, and female alates had more than male alates. The differences among cell numbers, however, were not significant (*F* = 1.20; df = 2; *P* = 0.331) (workers = 785 ± 165 [n = 7]; soldiers = 703 ± 155 [n = 6]; female alates = 687 ± 34 [n = 2]; male alates = 602 ± 148 [n = 2]).

### Other tissues

No immunostained cells or axons were observed in or associated with the fat body and Malpighian tubules of all castes or the testes and ovaries of two day and six day old male and female alates.

### Preabsorption controls

No immunoreactivity was observed in the nervous system or gut of the different castes when the antiserum was preabsorbed with *Hez* MP-I, *Ang* sNPF I, *Ang* NPF, or when the primary antibody was omitted. Preabsorbtion with FMRF-NH2 did not noticeably change immunostaining.

## Discussion

In this study, an NPF-like peptide was localized in numerous specific cells distributed in the both the central nervous system and gut of *R. flavipes* caste members. So distributed, release of this neuropeptide messenger likely integrates feeding behaviors regulated by the nervous system in relation to food content and its digestion and passage in the gut. These putative roles for NPF in termite digestion are supported by studies of dipterans for which NPF regulates feeding and food-searching behaviors (Shen and Cai 2001; Wu et al. 2003; Wu et al. 2005; Lingo et al. 2007) as well as midgut motility and ion transport (Onken et al. 2004).

The brain and ventral nerve cord of alate, worker and soldier *R. flavipes* contained similar distributions of cells and axons immunoreactive to *Hez* MP-I antibody. Immunoreactive axons leaving the brain entered the frontal ganglion or were associated with the corpora cardiaca/corpora allata complex and the foregut and midgut with branches extending over the surface of the salivary glands. Immunoreactive axons occurred on the rectum. Over 600 immunoreactive midgut endocrine cells were also observed in midguts with higher numbers occurring in the worker caste. The immunoreactive peptide in the central nervous system and midgut endocrine cells likely shares sequence similarity to other NPFs. The *Hez* MP-I antibody used for immunocytochemistry recognizes *Dm* NPF (Brown et al. 1999), and immunostaining in tissues was abolished when the antiserum was preabsorbed by *Hez* MP-I and *Ang* NPF and *Ang* sNPF-I. Together, these results indicate that the *Hez* MP-I antibody has specificity for peptides with an RXRF-NH_2_ C-terminus, a shared feature of these three peptides (Table 1). Immunostaining in termites was not changed when antiserum was preabsorbed with FMRF-NH_2_. This indicates that the *Hez* MP-I antiserum is specific for an NPF-like sequence and does not have a strong affinity to peptides with only an RF-NH_2_ C-terminus such as extended FMRF-NH_2_, myosuppressins, and sulfakinins.

Immunocytochemistry has been an important tool for describing NPY-related peptides in insects. Detailed immunocytochemical studies with NPF have so far only been reported for holometabolous insects, in particular *D. melanogaster* (Brown et al. 1999), *Ae. aegypti* (Stanek et al. 2002) and *H. zea* (Huang 1995). The brains of *Manduca sexta* also showed PP-like immunoreactivity in cells and in axons that led to the corpora cardiaca and the aorta (El-Salhy et al. 1983). Patterns of immunostaining in central nervous system and gut among these insects and *R. flavipes*were similar. Paired, immunoreactive, neurosecretory cells were observed in the brain of all of these insects. In *H. zea* and *R. flavipes,* immunoreactivity was observed in cells on the ventral nerve cord ganglia and axons on the surface of the corpora cardiaca. Also, the gut tract of *H. zea, Ae. aegypti,* and *R. flavipes* each exhibited immunoreactive axons at various points.

The distribution of NPF-like peptides in hemimetabolous insects has been described primarily from studies with antisera from vertebrate NPY-related peptides. Immunoreactivity to NPF and NPY antibodies has been reported in brain, subesophageal ganglion and gut of *P. americana* and *S. gregaria,* but a complete description of cell bodies or axon processes is lacking (Zhu et al. 1998). PP-immunoreactivity in *P. americana* (Endo et al. 1982a) and *S. gregaria* (Myers and Evans 1985) and NPY-immunoreactivity in *L. migratoria* central nervous system (Schoofs et al. 1988) was similar to *R. flavipes* NPF-like immunoreactivity, especially with regard to clusters of immunoreactive cell bodies in the central nervous system.

Midgut endocrine cells from several insects are reactive to NPF or NPY/PP antisera. NPF-like immunoreactive midgut endocrine cells in *R. flavipes* share similarities with those observed in *D. melanogaster* (Brown et al. 1999), *Ae. aegypti* (Stanek et al. 2002) and *H. zea* (Huang 1995), although the distribution and number of cells differed. NPY-like immunoreactive midgut endocrine cells have been similarly reported in *L. migratoria* (Schoofs et al. 1988) and *Tramea Virginia* (Patankar and Tembhare 2006). PP-immunoreactive midgut endocrine cells occur in *P. americana* (Iwanaga et al. 1981), *Gryllus bimaculatus* (Iwanaga et al. 1986), *Calliphora vomitoria* (Duve and Thorpe 1982) and *Ae. aegypti* (Brown et al. 1986).

The patterns of NPF immunostaining observed in the central nervous system and the gut among alates, workers and soldiers were similar suggesting these peptides may share similar functions in all three castes. NPFs share an RF-amide C-terminus with myosuppressins, extended FMRF-NH2, sulfakinins, sNPF and head peptides. Many of these RF-amides have myostimulatory or myoinhibitory effects on gut tissues (Fujisawa et al. 1993; Lange and Orchard 1997). Immunoreactive axons on the *R. flavipes* midgut suggest that they may have such a function allowing or preventing pumping of food through the gut. Rings of innervation at the junction of the crop, proventriculus, hindgut and rectum may also influence access of gut contents through gut compartments. Similar patterns of immunoreactivity have been observed in *Ae. aegypti* with other neuropeptides (Veenstra et al. 1995).

The diverse peptides expressed by endocrine cells in the insect midgut are thought to regulate different processes associated with digestion (Brown and Lea 1990; Veenstra et al. 1995). Leucomyosuppressin and Dippu-allatostatin 7 increase secretion of digestive enzymes in the midgut of the cockroach, *Diploptera punctata* (Fuse et al. 1999), and presumably this is mediated by such peptides originating from midgut endocrine cells. To date, there is no evidence that NPF affects digestive enzyme release in the insect midgut, but it does inhibit ion transport in the midgut of *Ae. aegypti* larvae (Onken et al. 2004), where it is present in midgut endocrine cells. NPF released from midgut endocrine cells into the hemolymph (Jenkins et al., 1989) would be carried to other tissues, including the nervous system, where it may stimulate or modulate processes associated with nutrient states. It has been suggested that the receptive termini of axons on the surface of the gut may be activated by peptides released by the midgut endocrine cells, bridging the gap between the gut and nervous system through the brain-gut axis (Sehnal and Zitnan, 1996). The higher number of immunoreactive midgut endocrine cells in worker termites may indicate that this communication plays a role in the differentiation of caste feeding behavior.

Regeneration of columnar and endocrine cells from nidi in the midgut tissue of cockroaches was noted long ago (Endo and Nishiitsutsuji-Uwo 1982b) and more recently reported in termites (Tokuda et al. 2001). We typically observed two or more endocrine cells immunoreactive to *Hez*-MP-I antisera within each nidal cluster, and as many as five were observed. Variation in number of midgut endocrine cells was observed in *L. migratoria,* where between zero and three endocrine cells were observed per regenerative niche (Illa-Bochaca and Montuenga 2006).

This study represents the first description of NPF-like immunoreactivity in termites. Mapping the distribution of an NPF-like peptide in the nervous system and midgut of *R. flavipes* was the first step to understanding its importance. We recently purified the *R. flavipes* NPF from a tissue extract with HPLC, as monitored with the *Hez* MP-I radioimmunoassay (unpublished results) and sequencing and molecular characterization revealed that it is an authentic NPF. Fractions containing this NPF accounted for most of the immunoreactivity in the extract, thus supporting the specificity of the *Hez* MP-I antiserum. The tissue distribution described herein will serve as a guide for the testing of synthetic NPF to discover its function in termites.

## Abbreviations

NPF: neuropeptide F
NPY: neuropetide Y
PP: pancreatic polypeptide
PYY: peptide YY
